# On the Clinical Use of Nuclein

**Published:** 1911-09

**Authors:** 


### On the Clinical Use of Nuclein.

Nuclein is a complex proteid body characterized by its large per-percentage of phosphorus. The phosphorus exists in the form of nucleinic acid. As far as is known, this acid is the same in all cells, although the basic part differs in various nucleins.

The therapeutic value of nuclein depends on two facts: First, upon its content in phosphorus, which is so necessary an element for the tissue metabolism; second, upon the fact that it is a normal constituent of all tissue cells, and that in disease this normal constituent is apt to be deficient. The therapeutic exhibition of nuclein then supplies the material with which to make good the deficit, and it is probably upon this basis that we must explain at least some of the excellent results that have been recorded from the use of the drug.

While nuclein has been obtained from a variety of sources—from yeast (by Miescher, Vaughan and others); from the thyroid and thymus glands; the spleen and other glands, it is for many reasons preferable to resort to the vegetable kingdom; to be exact, to the wheat germ, as a source of nuclein for therapeutic purposes, since it has been found that nuclein derived from animal sources is apt to develop toxic properties which have never been found in vegetable nucleins.

From the preceding, the indications for nuclein are primarily in all conditions of deficient vitality, such as anemias and the conditions of malnutrition due to faulty digestion. Owing to the very emphatic stimulating action of nuclein upon the number of leucocytes, more especially on the phagocytes, the drug has found its best known and best appreciated field of administration in conditions due to bacterial infection, and this was made use of successfully some years ago by Miculicz, for the prevention of operative infectious peritonitis. A number of very interesting communications have been published on this subject from his clinic in Breslau. All experiments, whether in the laboratory or clinical, show that the direct action of nuclein is exerted in the direction of producing hyperleucocytosis, the phagocytes being the cells particularly affected. It follows that nuclein is an efficient remedy to counteract not only infections proper, but also infectious diseases, at least in their early stages, and this point, suggested already by Vaughan in 1894, has been, in recent years, elaborated and proved clinically, more especially by Professor Ward of St. Louis, who communicated in January, 1910, a report to the Academy of Medicine in Chicago upon his results with intravenous injections of the drug made from the wheat germ. Since then the tritico-nucleinate of sodium has been used largely, orally, hypodermically and intravenously, and in many cases with the happiest results.

Nuclein is not a cure-all, nor can its germicidal powers be called more than slight, but it stimulates the resistance of the organism to bacterial infection and bacterial disease, it enables the organism, weakened from any cause whatever, to recuperate more rapidly than it would without the aid of this drug.

It has been found of particular value as a surgical dressing, undoubtedly owing to its stimulating effect upon the leucocytosis, and the uses of nuclein have become many and varied. It goes without saying that a dependable, uniform and standardized preparation should be used.

For full information on the tritico-nucleinate of sodium, write to the Abbott Alkaloidal Company, Chicago.

---

"Non-bacterial" pyuria is very suspicious of tuberculosis.—*American Journal of Surgery.*

For Pruritus Ani.—Irrigate daily with 1:1000 solution of quinine bisulphate and apply:

℞ Camphor .................................. 4 gr.
Menthol .................................. 3 gr.
Phenol .................................. ½ dr.
Boric Acid
Calomel..................................a a 10 gr.
Ointment of zinc oxide................q. s. ad 1 oz.

—*Therapeutic Medicine.*

